# Mules and Hinnies Weight Estimation Using Morphological Measures

**DOI:** 10.3390/ani16192985

**Published:** 2026-09-22

**Authors:** João B. Rodrigues, Stuart L. Norris, Katharina Habeck, Ahmed Khairoun, Claudia Valderrama, María Alejandra Torres-Medina, Juan Sebastián Galecio, Miriam Alva Trujillo, Sarah Worth, Holly A. Little, Tamara A. Tadich

**Affiliations:** 1 The Donkey Sanctuary, Sidmouth EX10 0NU, UK; stuart.norris@thedonkeysanctuary.org.uk (S.L.N.); sara.worth@thedonkeysanctuary.org.uk (S.W.); holly.little@thedonkeysanctuary.org.uk (H.A.L.); 2 Bundeswehr, 10785 Berlin, Germany; katharinahabeck@bundeswehr.org; 3 American Fondouk, Fez 30000, Morocco; ahmedkhairoun@gmail.com; 4 Departamento de Salud Animal, Facultad de Medicina Veterinaria y Zootecnia, Universidad Nacional de Colombia, Bogotá 111321, Colombia; ccvalderramam@unal.edu.co (C.V.); matorresme@unal.edu.co (M.A.T.-M.); 5 Escuela de Medicina Veterinaria, Colegio de Ciencias de la Salud, Universidad San Francisco de Quito, Cumbayá, Quito EC 170157, Ecuador; jgalecio@usfq.edu.ec; 6 Facultad de Medicina Veterinaria y Zootecnia, Universidad Veracruzana, Veracruz C.P. 91710, Veracruz, Mexico; mialva@uv.mx; 7 Instituto de Ciencia Animal, Facultad de Ciencias Veterinarias, Universidad Austral de Chile, Casilla 567, Valdivia 5090000, Chile; tamara.tadich@uach.cl

**Keywords:** equid hybrids, hinnies, mules, morphological measures, weight formula

## Abstract

Hybrid equids such as mules and hinnies play a critical role in supporting livelihoods in low- and middle-income countries but remain underrepresented in veterinary research. One key challenge is the absence of a practical and internally validated method for estimating body weight in mules and hinnies, which is essential for accurate dosing and management, particularly in the absence of a weighbridge. This study aimed to evaluate the performance of existing equid weight estimation models on mules and hinnies and to develop a new mules and hinnies -specific model to improve predictive accuracy and field usability. Three hundred and sixteen healthy mules and hinnies were assessed across nine countries. Sixteen morphological measurements were collected, and body weight was recorded using a digital weighbridge. Existing models were tested for their feasibility, and linear regression model was developed to predict mules and hinnies’ weighbridge body weight from morphological measurements. Model performance was assessed using repeated cross-validation and standard error metrics. Machine learning methods were used as a secondary analysis to explore feature importance. Morphological predictors showed strong associations with weighbridge weight. Linear models demonstrated reliable predictive performance, with low prediction error across weight ranges. The new mules and hinnies-specific model based on heart girth, chest width, and forearm girth, achieved an adjusted r^2^ of 0.94 (*p* < 0.001), providing estimates of live-weight without requiring classification by sex, conformation, or type. These findings demonstrate that simple morphological measurements can provide good estimates of body weight of mules and hinnies in field settings, greatly supporting the daily management of mules and hinnies in different contexts, and contribute to improve their health and welfare globally.

## Simple Summary

Working mules and hinnies are vital for supporting human livelihoods in developing countries, yet they are often overlooked in veterinary research. Knowing their exact weight is crucial for correct medicine dosing and proper care, but weighing scales are rarely available in the field. This study aimed to create a reliable and practical method to estimate the weight of these animals using simple body measurements. We examined three hundred and sixteen healthy mules and hinnies across nine countries, comparing their real scale weight with various body measurements. The results showed that measuring the animal’s heart girth, chest width, and forearm girth provides a highly accurate estimate of its real weight. Within the study population, the model performed consistently across sex and conformation categories. In conclusion, this simple measurement tool offers field workers an accessible way to monitor animal health and improve the welfare of working mules and hinnies worldwide.

## 1. Introduction

It is estimated that there are 7,887,306 of mules and hinnies worldwide, with 96% of the population present in Low and Middle-Income countries acting as working animals [1,2]. These figures illustrate their importance for millions of people within some of the world’s poorest communities [3,4], playing a key role as clean, accessible, and sustainable co-workers in sectors such as mining [5], tourism, riding and pack work in mountainous areas, construction [6,7,8], and agroforestry activities [9,10] across numerous contexts, contributing to resilience, productivity, economic growth, and diversification [11,12]. On a smaller scale, mules and hinnies also play an important role for leisure and sport activities, as companion animals [13], and for military and disaster relief purposes [3].

Despite their historical and contemporary importance, of mules and hinnies are by far the less studied of all domestic equids [13] with a direct impact on the resources available for both husbandry and management, and clinical care. The lack of a consistent method to estimate the body weight of mules and hinnies in the absence of a weighbridge is just an example, and the ability to do so is crucial to ensure e.g., accurate dosing of medications, proper feeding strategies, and appropriate workload management [5,14].

When a weighbridge is not available, body weight of equids can be estimated by using a weight tape [15], but existing ones are species-specific and developed for donkeys and horses, taking into consideration differences in anatomic conformation, body structure, and muscle and fat distribution [16]. Mules and hinnies possess unique structural and metabolic features that result in weight distribution that may not fully align with either parent species, and therefore the applicability of previously developed models for horses and donkeys and based on different methodologies including body measurements, body condition scoring, and statistical modelling [13,15,17,18,19,20,21] to hybrids remains uncertain, highlighting the need for mules and hinnies-specific weight estimation model. Furthermore, tools intended for use in field conditions must remain simple and practical, as complex measurement requirements may limit uptake among veterinary and paraprofessional practitioners. Therefore, this collaborative study evaluates morphological predictors of body weight in mules and hinnies and develop a practical weight estimation approach using field-collected measurements.

## 2. Materials and Methods

### 2.1. Study Setting and Animal Husbandry

Animals were maintained under their standard, real-world field conditions across all participating international locations, including military facilities, welfare sanctuaries, and private smallholder agricultural setups. To ensure minimal impact on their welfare, equids were kept loose in their normal environments and were only handled for the short period required to conduct the general health physical examination, take the body measurements and record weights. They returned to their normal routine immediately after the procedures were completed.

### 2.2. Sampling Rationale and Eligibility Criteria

A convenience sampling strategy was used to select the study cohort. All mules and hinnies included in this research were physically examined by experienced equid veterinarians, and only animals assessed as healthy were included. As mules and hinnies were recruited through convenience sampling and therefore may not represent the wider global population.

### 2.3. Data Collection

Data collected in the present study included individual data of each hybrid: mule or hinny; conformation (light/riding type, draft type animal or standard), type of use, age, body condition score, and sex; followed by sixteen morphological body measures; estimation of body weight using 2 species-specific tapes: the GSF Donkey weight tape company (Stratford-upon-Avon, Warwickshire, UK) and the Shires Equestrian Horse & Pony Weigh Tape 838 Weighing Weigh band-One Size (Leominster, Herefordshire, UK); and obtaining body weight using a digital weighbridge.

Data collection kit

Data collection kit used in the present study included an electronic device with the android operating system for ODK (Get ODK Inc., San Diego, CA, USA) [17] app use, a standard measuring stick (Lincoln Horse Height Measuring Stick, Battle, Hayward & Bower Ltd., Lincoln, Lincolnshire, UK), a conventional Shires Equestrian metallic measuring tape, a Shires Equestrian flexible measuring tape, and a zero-point extension for the measuring stick-a 30 cm plastic extension specifically developed for this project and printed in a 3D printer, converting it in a “U” shape gauge, but also used alone as a landmark for other body measurements.

Data collection app

Data was collected using a bespoke ODK collect app [17], chosen due to its flexibility and ability to collect data in offline areas, while minimising transcription errors and data lose. An ODK form was designed to collect all parameters described below, and once collected data was sent via the internet to ODK central server.

Data collectors’ selection and training

All data collectors involved in this research are veterinarians and/or researchers with experience in dealing with mules and hinnies and were invited to participate due to their access to healthy animals, and the ability to collect large sets of data.

To reduce interobserver variation training was provided to each data collector using a virtual learning environment, throughout a mandatory online course with detailed videos covering in depth all aspects of data collection, including installation of ODK app, and specific details of the method required for each individual measurement. A data collection kit was sent to all data collectors (except for the android device), ensuring data collection consistency and reliability.

### 2.4. Morphological Measures

**Figure 1:**
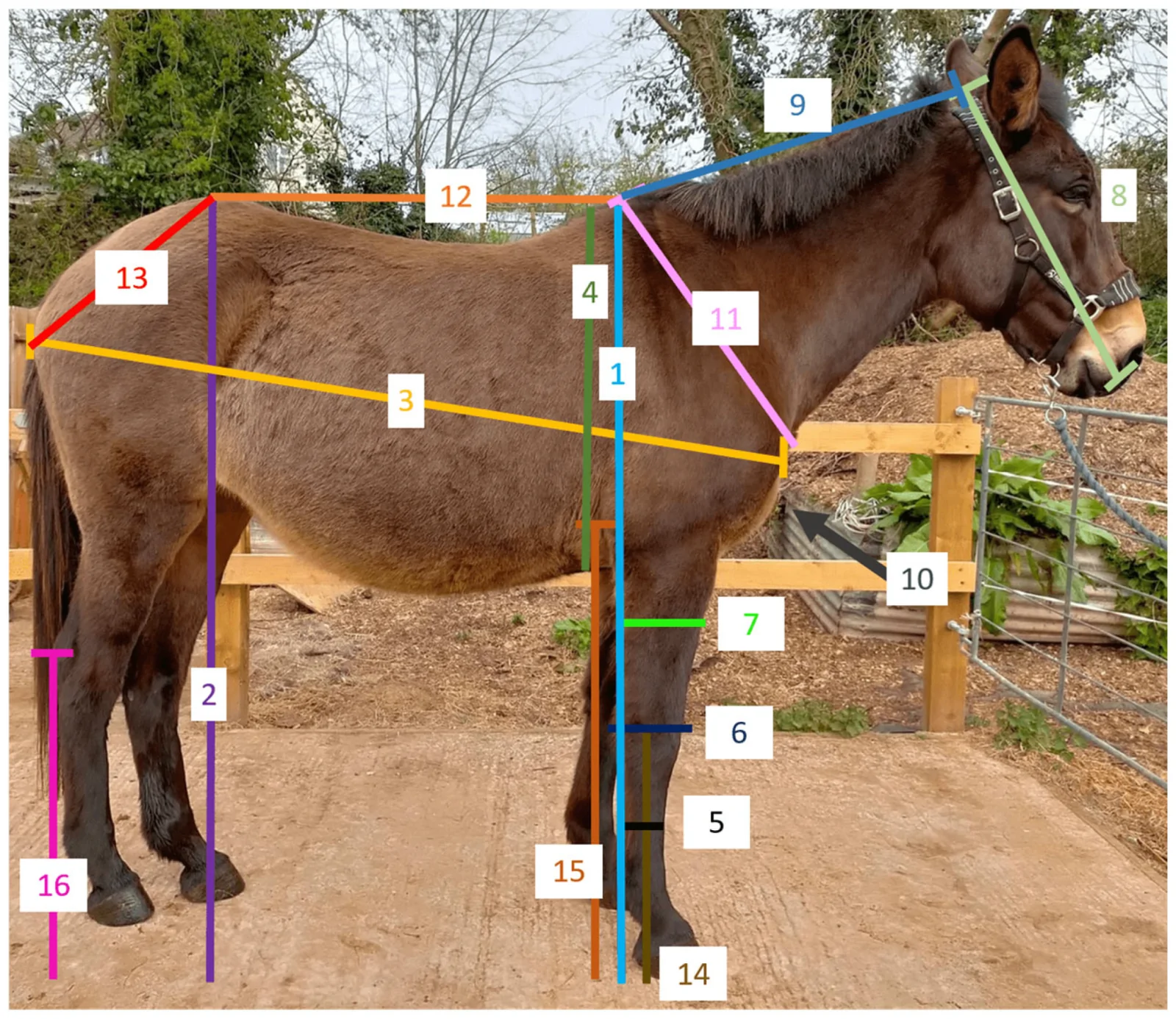
Morphological measures collected for each animal involved in the study. 1: Height. 2: Rump height. 3: Body length. 4: Heart girth. 5: Cannon bone girth. 6: Knee girth. 7: Forearm girth. 8: Head length. 9: Neck length. 10: Chest width. 11: Shoulder length. 12: Back length. 13: Rump length. 14: Knee height. 15: Elbow height. 16: Hock height.

Sixteen morphological measures were collected for each mule and hinny, with the animals kept square on a level surface (Figure 1). To ensure data consistency and minimize handling bias, all 16 measures were taken in the exact same predefined order across all 316 animals.

Morphological measures were obtained in centimetres based on the measurements used by Lagos et al. [18], as described below:

1. Height: Using the measuring stick, measure from the ground to the highest point of the withers.
2. Rump height: Using the measuring stick, measure from the ground to the point of croup.
3. Body length: Using the measuring stick in combination with the zero-point extension, measure from the head of the humerus to the point of buttock (*tuber ischii*). Make sure the that lower part of the measuring stick fully enters the socket on the extension, and that it is securely tightened. While measuring, the extension stays in contact with the point of buttock, while the movable arm of the measuring stick slides in front of the animal at the point of the shoulder level. Use two people if required.
4. Heart girth: Pass the flexible tape measure 10 cm behind the point of the withers and around the equids barrel. Make sure the ventral part of the tape is as close to the forelegs as possible and is stand in a vertical position when viewed from the side.
5. Cannon bone girth: Find the halfway point on the cannon bone between the carpus and the fetlock and measure the circumference of the cannon bone using the flexible measuring tape.
6. Knee girth: Measure the circumference of the knee joint in line with and including the accessory carpal bone using the flexible measuring tape.
7. Forearm girth: Measure the circumference of the forearm, midway between the knee and the elbow, using the flexible measuring tape.
8. Head length: Measure the head from the front of the muzzle to the top of the poll. To avoid the natural curvature of the muzzle region, please measure the head length in its lateral aspect, as this surface is flatter. Place the measuring tape in the top part of the neck, immediately behind the basis of the ear. Run the along the face to the ventral tip of the lips.
9. Neck length: Measure the distance between atlas vertebra and thoracic vertebra (distance between poll to withers), using the rigid measuring tape.
10. Chest width: Measure from point of shoulder to point of shoulder using the rigid measuring tape.
11. Shoulder length: Find the rounded edge of the top of the shoulder blade just below either side of the withers. Then measure down to the depression before the head of the humerus, using the rigid measuring tape.
12. Back length: Measure from the point of withers to the sacral tuberosity/point of the croup using the rigid measuring tape.
13. Rump length: From the point of buttock to point of croup, using the rigid measuring tape. To avoid the interference of the round shape of the croup, place the extension on the top of the croup, making sure that is parallel to the ground, that it makes a 90 degrees angle with the vertebral column of the mule, and that the back part of the extension is aligned with the point of the croup. Place the beginning of the measuring tape in contact with the extension and extend the tape until this is in line with the point of buttock.
14. Knee height: Measure from the ground to the point of the accessory carpal bone, in the palmar aspect of the foreleg, using a rigid measuring tape.
15. Elbow height: Measure from the ground to the point of the olecranon, in the palmar aspect of the foreleg, using the rigid measuring tape.
16. Hock height: Measure from the ground to the point of the hock, in the plantar aspect of the hindleg, using the rigid measuring tape.

### 2.5. Estimation of Body Weigh Using Species-Specific Tapes

After completion of the 16 body measurements, the heart girth of each individual animal was measured using the GSF Donkey weight tape company and the Shires Equestrian Horse & Pony Weigh Tape 838 Weighing Weigh band-One Size by following the same method described earlier for measurement 4, and the corresponding body weight registered.

### 2.6. Obtaining Body Weight Using a Digital Weighbridge

Each individual mule and hinny was weighed in kg using a digital weighbridge (Horse Weigh, Llandrindod Wells, Powys, UK), calibrated at the beginning of the study, and recalibrated after every tenth animal weighed to correct for any drift.

### 2.7. Existing Models

Existing models were tested for their effectiveness and accuracy in predicting mules and hinnies’ body weight (kg). For all the models the resulting body weight was rounded to 0 decimal places to align with the accuracy of the weighbridge used to obtain actual body weight. The formulas are detailed below Equation (1) [15], Equation (2) [19], Equation (3) [20], Equation (4) [21], Equation (5) [22], Equations (6) and (7) [23], Equation (8) [16]:

(1) $$\mathrm{BW}\left(\mathrm{kg}\right)=0.000252\times \left({\mathrm{HG}}^{2.576}\times H^{0.24}\right)$$

(2) $$\mathrm{BW}\left(\mathrm{kg}\right)=\frac{HG^{2.12}\times H^{0.688}}{3801}$$

(3) $$\mathrm{BW}\left(\mathrm{kg}\right)=0.021179\times {\mathrm{HG}}^{1.81247}$$

(4) $$\mathrm{BW}\left(\mathrm{kg}\right)=3.339\times \mathrm{HG}+3.768\times \mathrm{BL}$$

(5) $$\mathrm{BW}\left(\mathrm{kg}\right)=0.00002\times \left(H^{0.122062}\times HG^{3.14841}\right)$$

(6) $$\mathrm{BW}\left(\mathrm{kg}\right)=\sqrt{112.05}+H^{2.24}$$

(7) $$\mathrm{BW}\left(\mathrm{kg}\right)=\sqrt{49.997}+H^{1.78}$$

(8) $$\mathrm{BW}\left(\mathrm{kg}\right)=-332+2.75\times \mathrm{HG}+1.44\times \mathrm{BL}$$

(9) $$\mathrm{BW}\left(\mathrm{kg}\right)=-357.36+\left(2.89\times \mathrm{HG}\right)+\left(0.86\times H\right)+\left(0.58\times \mathrm{BL}\right)+\left(-0.03\times \mathrm{HNL}\right)+\left(2.09\times \mathrm{CBG}\right)$$

(10) $$\mathrm{BW}\left(\mathrm{kg}\right)=-359.13+\left(2.72\times \mathrm{HG}\right)+\left(0.86\times H\right)+\left(0.55\times BL\right)+\left(-0.03\times HNL\right)+\left(3.13\times \mathrm{CBG}\right)$$

(11) $$\mathrm{BW}\left(\mathrm{kg}\right)=-359.20+\left(2.81\times \mathrm{HG}\right)+\left(0.82\times H\right)+\left(0.58\times \mathrm{BL}\right)+\left(-0.04\times \mathrm{HNL}\right)+\left(2.12\times \mathrm{CBG}\right)$$

(12) $$\mathrm{BW}\left(\mathrm{kg}\right)=-359.36+\left(2.81\times \mathrm{HG}\right)+\left(0.82\times H\right)+\left(0.5893\times \mathrm{BL}\right)+\left(-0.03\times \mathrm{HNL}\right)+\left(2.09\times \mathrm{CBG}\right)$$

(13) BW (kg)=−356.04+(2.80×HG)+(0.83×H)+(0.57×BL)+HNL+(2.01×CBG) 

Letters in the equations represent body weight (BW), height (H), heart girth (HG), body length (BL), cannon bone girth (CBG), head and neck length (HNL). All measurements were in centimetres except body weight which was in kg.

Formula (6) relates to male donkeys and Formula (7) relates to female donkeys, both were included to see how well the relate to body weight of mules and hinnies. Formulas (9)–(13) are derived from existing data held by The Donkey Sanctuary (TDS) on donkey physiology and were also tested in the present study. Models 9, 10 and 11 were derived using a simple linear regression, 12 was derived using a Bayesian model, and 13 was derived using a Generalized Linear Models (GLM) net model.

### 2.8. Statistical Analysis

All statistical analyses were performed using R 4.2.1 [24] in RStudio v2022.07.2-576 (Posit Software, PBC, Boston, MA, USA) [25]. The tidyverse package v2.0.0 (Posit Software, PBC, Boston, MA, USA) [26] was used for data transformation and visualisation. Additional statistical analyses were conducted using the Car package v3.1-5 (Fox & Weisberg, 2019; McMaster University, Hamilton, ON, Canada) [27] for regression diagnostics, MASS v 7.3-66 (Venables & Ripley, 2002; University of Oxford, Oxford, Oxfordshire, UK) [28] for model selection, and tidymodels v1.5.0 [29] for machine learning and predictive modelling.

Descriptive statistics were applied to examine the distribution of sex, conformation, usage, and geographical origin of mules and hinnies. The data were summarised using means, standard deviations, and frequencies to provide an overview of the sample population. Understanding these baseline characteristics also allowed for more informed comparisons when evaluating existing weight estimation models.

Existing models and linear regression models were developed to predict weight from morphological predictors. Model performance was evaluated using repeated k-fold cross-validation. Prediction accuracy was assessed using mean absolute error (MAE), root mean square error (RMSE) and mean absolute percentage error (MAPE).

Regularised regression models were explored as robustness checks. Gradient boosting methods were applied as a secondary analysis to assess feature importance but were not used as the primary predictive model.

Feature selection was conducted as an exploratory initial step using XGBoost v3.2.1.1 (DMLC, University of Washington, Seattle, WA, USA) to identify the most influential morphological predictors within the training dataset. XGBoost was used solely as a screening tool to identify candidate predictors and was not used as the final predictfive model.

XGBoost was used only as an exploratory screening procedure, and the final model was deliberately restricted to three biologically plausible predictors.

Following feature identification, the final multivariable linear regression model was fitted, and its predictive performance and generalizability were independently evaluated using repeated k-fold cross-validation.

While feature selection and final model evaluation were conducted sequentially, predictor selection was not repeated within each cross-validation fold. Consequently, the reported cross-validation statistics should be interpreted as internally validated performance estimates and may retain a degree of optimism. However, the final model was deliberately restricted to three biologically plausible predictors to reduce overfitting and improve interpretability.

## 3. Results

### 3.1. Data Summary

A total of 316 adult mules and hinnies (median of 14 yo-min = 3; max = 36) were assessed as part of this research, comprising 96% mules (*n* = 304) and 4% hinnies (*n* = 12) (see Table A1). Among the assessed equids, 53% were female and 47% were male, reflecting a relatively balanced distribution of sexes. All equids included in the study presented a body condition score between 3 (ideal) and 4 (overweight), based on the donkey body condition score chart developed by The Donkey Sanctuary that uses a 1-to-5 scale to evaluate muscle and fat coverage through hands-on feeling [30]. All males included in this study were geldings, and all females were empty/non-pregnant. In terms of conformation, 21% were classified as draft-type, known for their heavier build and suitability for carrying/pull substantial loads, 27% had a light conformation, typically used for riding and agility-based work, while 51% exhibited a standard conformation, representing the general working hybrid population.

Regarding their functional roles, 72.2% were utilised in military or army settings both as riding and pack animals, highlighting their importance in transport and logistical support in various terrains; 10.8% were housed in sanctuaries, where they were primarily rescued or retired working animals; 4.1% were employed for pleasure riding, while 12.9% were working equids used as pack animals, for agroforestry activities as traction animals, and cart-pulling tasks.

The study encompassed nine countries across Europe, Central and South America, and Northern Africa, providing a broad geographical representation of mules and hinnies’ usage (Table 1).

### 3.2. Effectiveness and Accuracy of Existing Models

Eight existing models developed for weight estimation of equids were tested using the dataset collected in the present study, in predicting body weight (kg) of mules and hinnies.

Results are presented in Table 2 and indicate that the models by Cherdchutham [21] and De Aluja [22] demonstrated the highest accuracy when applied to mules and hinnies, with mean differences of 30.1 kg and −7 kg, respectively, and the lowest MSE and MAE values among the models tested.

Conversely, the model developed by Noikhrua [20] performed the worst, with substantial underestimations of body weight by an average of −156.4 kg, with an MSE of 29,381.50, demonstrating that this model is not suitable for accurate weight estimation in mules and hinnies.

Models derived from an internal dataset of donkey morphological measurements held by TDS performed relatively well in predicting weight of mules and hinnies. Among these, the TDS Linear 2 model (model 10 in the list of equations) showed the best performance, with a mean difference of −28.4 kg and an MSE of 3713.1, indicating that a linear regression approach applied to donkey-specific data can be effectively adapted for mules and hinnies weight estimation. In contrast, the TDS GLM model (model 13) performed the worst among The Donkey Sanctuary models, with a mean difference of 104.2 kg and an MSE of 13,705.3, suggesting that generalised linear models (GLMs) may not be as well-suited to mules and hinnies’ weight prediction as simpler linear regression approaches.

Model comparisons to actual body weight (Table 2) indicate that certain models, such as Noikhrua [20] and John (Female) [23], significantly underestimate weight, making them unsuitable for mules and hinnies. In contrast, models like De Aluja [22], Cherdchutham [21], Pearson [19], the Horse weight tape and TDS Linear 2 performed well, as their mean differences are closer to zero, suggesting minimal bias. Models where the weight is greater than 0 (TDS GLM, Cherdchutham [21], Pearson [19] and Eley [15]) tend to overestimate weight, though their errors are relatively small compared to the most underestimating models.

Among the TDS models (Table 2), the TDS Linear models outperform the TDS GLM, suggesting that linear regression-based approaches are more effective for weight estimation of mules and hinnies compared to generalized linear models. The combination of these results with MSE highlights the high predictive accuracy of linear regression models overall. Furthermore, analyses using MSE and MAE indicate that linear regression models do not systematically overestimate or underestimate weights of mules and hinnies, unlike Bayesian and Generalized Linear models.

**Figure 2:**
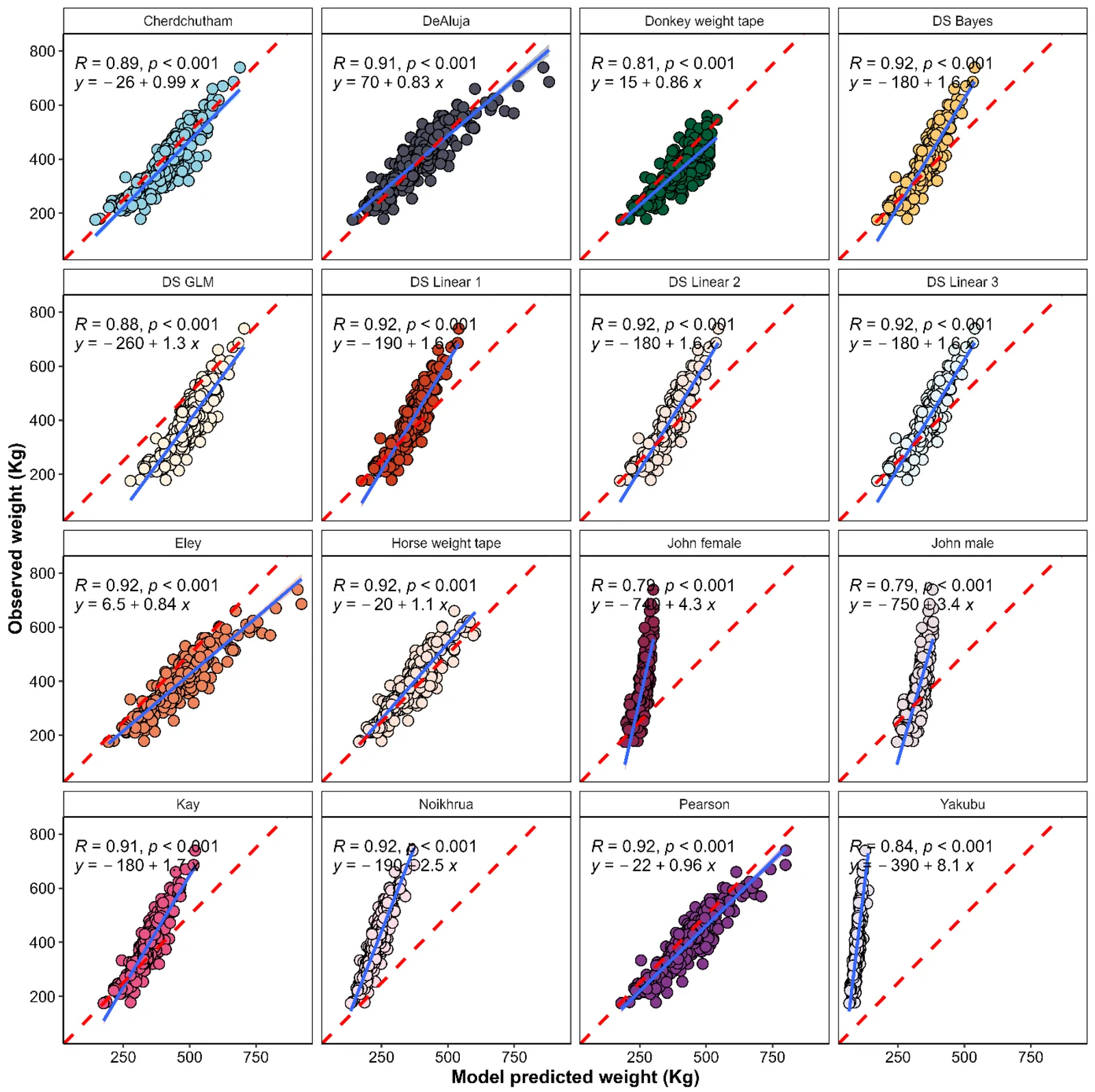
Comparison of each model’s predicted weight (kg) to observed body weight (kg) recorded using a weighbridge. Each panel represents a different model, with the correlation coefficient (R), *p*-value, and linear regression equation provided. The red dashed line indicates a perfect 1:1 relationship, while the blue regression line shows the actual fit of the model to the data. Models with slopes closer to 1 and minimal intercept biases indicate better predictive performance.

Figure 2 provides a visual evaluation of the predictive performance of various models by comparing their predicted weights to observed body weights. Models with higher correlation coefficients (R values) and regression slopes closer to 1 demonstrate stronger predictive accuracy.

Among the models, the horse weight tape, TDS Bayes, TDS Linear models (1, 2, and 3), Eley [15], and Pearson [19] each displaying r^2^ = 0.92, suggesting strong linear associations with body weight. The regression slopes for these models ranged between 0.84 and 2.5, indicating some systematic bias in weight estimation. Notably, the Cherdchutham [21] model had a slope closest to 1, indicating predictive reliability, but with a slightly lower r^2^ = 0.91 but was better at estimating heavier mules than the Eley [15] model.

Conversely, models such as John (Female) and John (male) [23] showed weaker performance, with lower R values (0.79) and greater deviations in slope from the ideal value of 1. The John (female) model exhibited the largest bias, with substantial underestimations of body weight, as indicated by their low slopes (4.3) and large positive intercepts.

Overall, the results suggest that TDS Linear models and Cherdchutham [21] are among the most accurate approaches for weight estimation of mules and hinnies, whereas models like John (female) [23] introduce significant prediction errors, likely due to dataset-specific biases or differences in the weight distribution of the training data.

Table 3 provides additional insights into model performance beyond the correlation plots by quantifying systematic prediction errors and evaluating statistical significance through T-tests. The results indicate that the TDS Linear models, Eley [15], and Pearson [19] exhibit the highest correlation with body weights, with r^2^ values around 0.92, demonstrating strong predictive power. These models have relatively low residual values, ranging from −33.79 to 66.98 kg, meaning their predictions are more balanced and do not drastically over- or underestimate weights. However, the TDS Linear models tend to slightly underestimate weights, with residuals between −33 and −28 kg, whereas Cherdchutham [21] with the slightly lower r^2^ overestimates by 30.08 kg.

In contrast, Noikhrua’s [20] model perform the worst, with large negative residuals of −156 kg, indicating a substantial underestimation of body weight. Similarly, John (female) [23] significantly underestimates weights, with a residual of −122.47 kg. These models also have slightly lower r^2^ values (0.89–0.93), suggesting a weaker predictive relationship with body weight. In terms of bias, TDS GLM and Eley [15] consistently overestimate weights, with large positive residuals between 104.12 and 66.98 kg, and highly significant T-values. Conversely, TDS Bayes, and Noikhrua [20] systematically underestimates weights, as reflected in their strongly negative residuals. Using T-test comparisons demonstrates (Table 3) that the TDS Linear models and Cherdchutham [21] provide the most balanced predictions, while Noikhrua [20], and John (female) [23] exhibit significant underestimation. Although models like TDS GLM and Eley [15] have high r^2^ values, their tendency to overestimate body weight makes them less trustworthy for practical applications.

### 3.3. New Specific Method for Mules and Hinnies

Hyperparameter tuning was conducted across a range of tree architectures, learning rates, and tree depths (Table 4), with model performance assessed using 10-fold cross-validation. The objective of this analysis was not to develop the final predictive model, but to identify the most influential morphological predictors of body weight. The optimal XGBoost configuration consisted of 1567 trees, a tree depth of 8, a minimum node size of 3, and a learning rate of 0.008. Variable importance derived from this tuned model identified heart girth, forearm girth, and chest width as the strongest predictors, which were subsequently incorporated into the final multivariable linear regression model.

**Figure 3:**
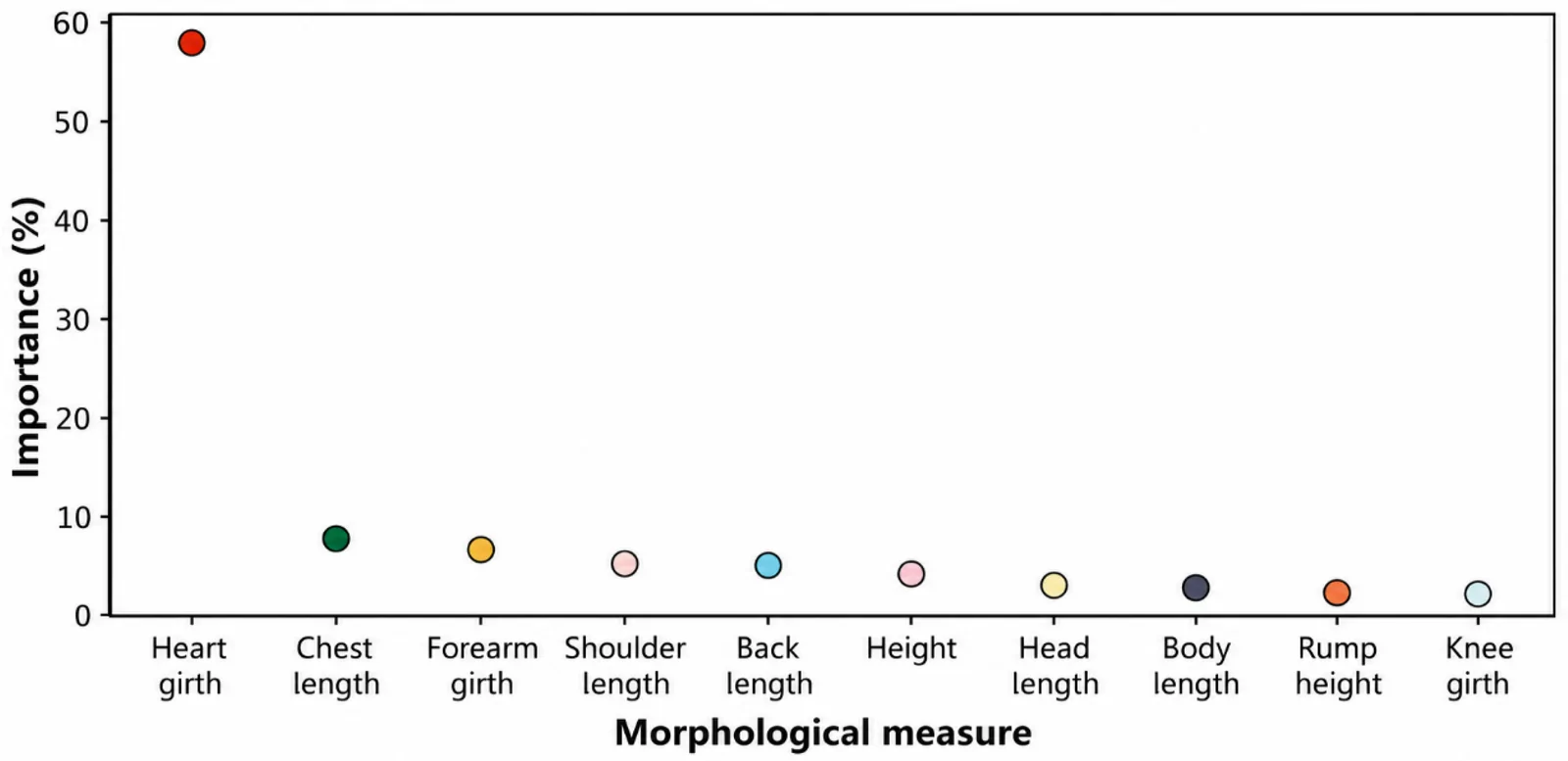
Relative importance of the top 10 morphological measures in predicting body weight (kg) of mules and hinnies, based on the best-performing XGBoost sub-model. Heart girth, forearm girth, and chest width were identified as the most influential predictors, contributing the highest proportion of variance explained in the model. These findings informed the development of a simplified regression-based equation for practical weight estimation in field conditions.

Figure 3 provides insight into the factors influencing weight prediction by highlighting the morphological measures identified as most important by the XGBoost model. Heart girth emerged as the dominant predictor, contributing 58% to the model’s accuracy, emphasising its well-established correlation with body weight across equid species. Forearm girth and chest width were also key contributors (with 9% and 7%, respectively), reflecting structural elements that influence overall body mass.

Interestingly, head length (2%) and body length (3%) had lower importance scores but still contributed to the model’s predictive ability, suggesting their relevance in distinguishing conformation differences.

Based on the results from the XGBoosted model, the top three morphological parameters influencing body weight based on permutation importance are heart girth, forearm girth, and chest width, which together explain a significant proportion of the variation in weight. Leveraging these insights, we developed a linear regression model to provide a practical weight estimation method for estimating weight of mules and hinnies without requiring access to a weighbridge.

Compared to traditional weight estimation formulas, which often rely solely on heart girth and may not generalise well to mules and hinnies, this model incorporates additional key measurements. By doing so, it improves precision while maintaining ease of implementation, making it a valuable advancement in equid weight estimation.

$$\mathrm{Weight}\left(\mathrm{kg}\right)=-652.4\;+\;5.1\;\times \;\mathrm{Heart}\;\mathrm{Girth}\;(\mathrm{cm})\;+\;3.7\;\times \;\mathrm{Forearm}\;\mathrm{girth}\;(\mathrm{cm})\;+\;1.2\;\times \;\mathrm{Chest}\;\mathrm{width}\;(\mathrm{cm})$$

Model comparison demonstrated that adding additional demographic or anatomical covariates provided minimal improvements in predictive accuracy over the base morphological model. Specifically, comparing the base model (Model 1: heart girth, forearm girth, and chest width) against models incorporating sex (Model 2), type (Model 3), or conformation (Model 4) showed minimal changes in Root Mean Square Error (Model 1 RMSE = 30.4 kg, Model 2 ΔRMSE = −0.4%, Model 3 ΔRMSE = −0.4%, Model 4 ΔRMSE = −1%). Because the inclusion of type or conformation yielded no meaningful reduction in prediction error, Model 1 was retained as the final model, offering field usability without sacrificing accuracy. Geographic diagnostic modelling confirmed that country of origin also had a negligible impact on predictive accuracy. The country-level random effect variance was near zero ($\sigma_{\mathrm{Country}}^{2}<0.001$, SD<0.001), and a likelihood ratio test (χ^2^ = 0.48, *p* = 0.42) confirmed no statistically significant improvement over the standard fixed-effects linear regression model ($\chi^{2}\left(1\right)=0.00,\;p=1.000$). Fixed-effect parameter estimates and standard errors for heart girth, forearm girth, and chest width were identical to four decimal places between both models, demonstrating that morphological relationships with body weight are consistent across geographic regions.

**Figure 4:**
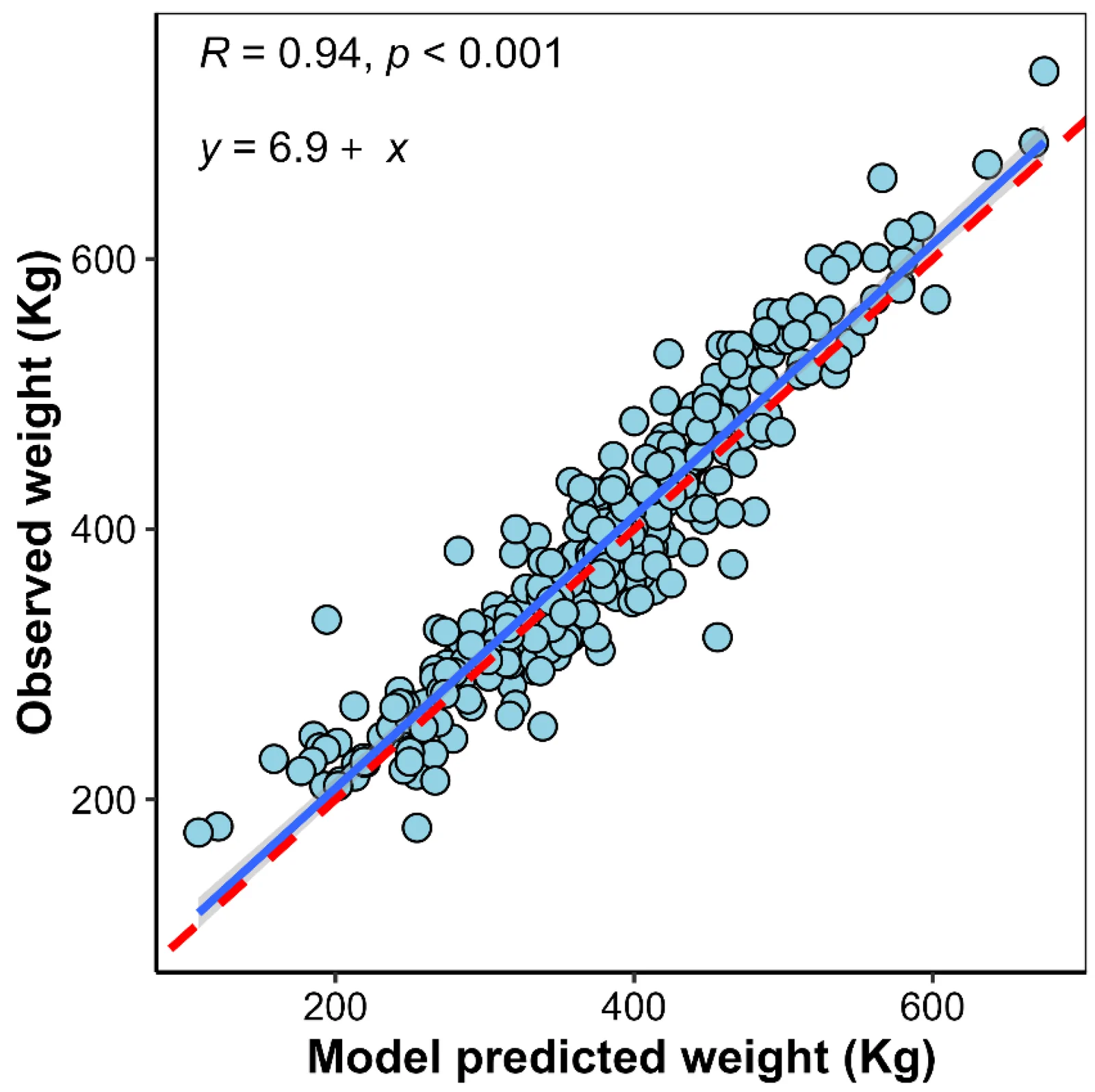
Linear regression model for predicting body weight (kg) of mules and hinnies using the most influential morphological features identified through the XGBoost model. The red dashed line represents a perfect 1:1 relationship between predicted and actual weight, while the blue regression line illustrates the model’s fitted relationship to body weight measurements. The shaded area represents the confidence interval, indicating the variability and uncertainty in predictions. This model, based on heart girth, forearm girth, and chest width, provides a practical alternative to weighbridge measurements, allowing for accurate weight estimation in field conditions.

Within the study population, the newly developed model demonstrated strong predictive performance relative to the alternative models evaluated, explaining 94% of the observed variation in body weight (Figure 4). Specifically, the final model demonstrated high precision with a Mean Absolute Error (MAE) of 23.4 kg, a Root Mean Square Error (RMSE) of 30.4 kg, and a Mean Absolute Percentage Error (MAPE) of 6.6%. However, these performance estimates should be interpreted as internally validated metrics because predictor selection preceded model evaluation. Despite this, the low relative gap between the RMSE and MAE indicates that the model’s predictions are consistently accurate without being severely skewed by extreme outliers. Furthermore, the model exhibited negligible systemic Bias −3.77 kg, confirming that it does not systematically over-predict but does marginally under-predict weight. Collinearity diagnostics also confirmed low variance inflation across predictors (heart girth VIF=3.9, forearm girth VIF=3.8, chest width VIF=3.4), indicating reliable coefficient estimates without multicollinearity distortion. Residual analysis also confirmed consistent predictive performance across the entire weight spectrum. Crucially, the model maintained high accuracy at the weight extremes, yielding a Mean Absolute Error of 28.4 kg for the lowest 10th percentile and 27.9 kg for the highest 90th percentile of body weights.

In contrast models such as Noikhrua [20] performed poorly, with large residual errors and systematic underestimation of weight, while even stronger models like Cherdchutham [21] and De Aluja [22] did not achieve the same level of explanatory power as the new model. Many of the models tested in this study relied heavily on heart girth alone, leading to systematic errors at the upper and lower weight extremes. By incorporating chest width and forearm girth, the new model accounts for additional structural factors that influence body weight, reducing bias and improving reliability across a wider range of mules and hinnies.

## 4. Discussion

Being able to correctly estimating the body weight of mules and hinnies is essential for appropriate veterinary care, nutritional management, and workload assessment [31]. Examining the predictions at the extremes of the body weight scale, it is evident that some models systematically over- or underestimate weights at the lower and upper ends. Models such as John (Male) [23] exhibit notable deviations at lower weights, as indicated by their relatively high intercept values (e.g., John (Male): intercept = 810) and slopes significantly different from 1.

Conversely, models like De Aluja [22] tend to underestimate heavier animals, with slopes less than 1 (De Aluja: 0.84), indicating that as body weight increases, the predicted values fall short of actual weights. This pattern suggests that these models may struggle with higher-weight mules and hinnies, possibly due to limitations in training data representation or the underlying assumptions of the model.

In contrast, models by Cherdchutham [21] and Pearson [19] demonstrate a more balanced performance across the entire weight range. Their slopes are closer to 1 (between 0.98 and 1.1), and their intercepts are relatively small, suggesting that they do not systematically overestimate lighter animals or underestimate heavier ones. Overall, the Cherdchutham [21] model emerge as the most interesting existing model due to the strong correlation with body weight (r^2^ = 0.91) and relatively small residual errors compared to other methods.

However, existing weight estimation models have largely been developed for either donkeys or horses, without consideration of the morphological differences that arise in hybrids such as mules and hinnies [20,21,32]. Many of the models assessed in this study, exhibited substantial prediction errors, with large residuals and systematic underestimation of body weight. Similarly, weight tape-based methods, such as those developed for donkeys and horses, often failed to generalise to their hybrids, leading to either overestimation or underestimation depending on the conformation of the individual animal (Table 2). These results align with previous research indicating that mules and hinnies cannot be reliably assessed using models designed for their parent species, as their unique morphology results in different weight distributions compared to donkeys and horses [33,34].

While some existing models, such as Cherdchutham [21] and De Aluja [22], performed relatively well, they still rely too heavily on a single predictor, most often heart girth, which, while strongly correlated with weight, does not fully account for variations in body structure [15]. This is particularly relevant for mules, which inherit the compact but muscular build of donkeys and the long-limbed proportions of horses, leading to weight distributions that are not well represented by single-variable models.

To address the limitations of existing models, this study developed a new mules and hinnies-specific weight estimation method by identifying the most relevant morphological predictors, followed by a regression-based approach to create an easily applicable predictive model. This process revealed that heart girth, forearm girth and chest width were the most influential predictors, which were then incorporated into a multiple regression model to improve accuracy.

The strong weighting of heart girth aligns with existing research but also demonstrates the advantage of incorporating additional measures, such as forearm girth and chest width, which might be underutilised in traditional weight estimation formulas. This highlights the potential of mules and hinnies-specific models to improve accuracy by adapting to the unique morphology of mules and hinnies, ensuring more precise weight assessments for better health and management.

Within the study population, the final model achieved an adjusted r^2^ value of 0.94, representing strong predictive performance relative to the other models evaluated. Unlike previously tested models, which often required additional data regarding type or conformation, this model does not require such classifications, making it more accessible and easier to use in field conditions. This is particularly important in low-resource settings where weighbridges and other precise measuring tools may not be available. By providing a model that can be applied using only three straightforward body measurements, this method offers a practical solution for veterinarians, para-veterinarians, and equid owners working in rural or low-income regions, where mules and hinnies play a crucial role in transportation, agriculture, and livelihoods [35]. However, performance in hinnies requires further validation due to the limited sample size (*n* = 12) and the model was developed using mules and hinnies ranging from 175.5–739 kg bodyweight, heart girth 125–220 cm, forearm girth 24–52 cm and chest width 21–50 cm. As such predictions outside these observed ranges should be interpreted with caution.

A limitation of this study relates to the morphological predictors being initially identified using XGBoost and subsequently incorporated into the final linear regression model. Although feature selection was undertaken within the training dataset and the final model was restricted to three biologically plausible predictors, predictor selection was not repeated within each cross-validation fold. Consequently, the reported performance metrics may slightly overestimate predictive performance in completely independent datasets. External validation in additional mule and hinny populations would provide a more rigorous assessment of generalisability and model stability.

The importance of an accurate weight estimation model extends beyond basic management practices, as incorrect weight assessments can lead to inaccurate medication dosages, inappropriate feed allocations, and potential welfare concerns [22]. In particular, working mules and hinnies in low-income countries are often subject to high workloads with limited veterinary oversight, making proper weight estimation crucial for ensuring appropriate healthcare interventions [34]. The ability to use a simple three-variable equation to estimate weight provides a practical solution for improving equid welfare in these settings.

This study’s hybrid approach, combining machine learning for feature selection with traditional regression modelling, ensures that the final weight estimation formula is both data-driven and interpretable. Unlike black-box machine learning models that may lack transparency in their decision-making processes, this method maintains a balance between complex data processing and field usability. By refining weight estimation methods specifically for mules and hinnies, this research contributes to a more accurate, accessible, and welfare-focused approach to working equids management.

## 5. Conclusions

This study has demonstrated that existing weight estimation models, originally designed for donkeys or horses, often fail to generalise to mules due to their unique morphological characteristics, either systematically under or overestimating weight of mules and hinnies. Among the existing models some performed relatively well, but they still lacked the precision required for accurate veterinary application.

Building upon these findings, this study developed a new mules and hinnies-specific weight estimation model. Using heart girth, chest width, and forearm girth, the final model achieved an adjusted r^2^ value of 0.94 within the study population, explaining over 94% of the observed variation in weigh while remaining simple and practical for field use. Unlike previous models, it does not require classification by type, sex or conformation, making it particularly valuable in resource-limited environments where precise weighing equipment is unavailable. This advancement has significant implications for veterinary care, particularly in low-income countries, where mules play a critical role in transport, agriculture, and labour-intensive industries such as brick kilns and mountainous pack transport [6]. Future research should focus on further refining this model by incorporating larger datasets, additional morphological measures, and external validation across diverse mules and hinnies’ populations, ensuring its continued reliability and applicability worldwide.

## Figures and Tables

**Table 1:** Countries where morphological characteristics were measured.

Country	Number of Mules	Number of Hinnies	Total
Chile	189	5	194
Germany	33	1	34
Spain	17	3	19
Morocco	19	0	20
Colombia	13	0	13
Italy	9	2	11
Ecuador	11	0	11
Mexico	11	0	11
United Kingdom	2	1	3
Total	304	12	316

**Table 2:** Comparison of each model and measuring tapes to body weight (kg) recorded using the weighbridge with number of observations, mean and lower (LCI) and upper (UCI) confidence intervals, the standard error mean squared error (MSE) and mean absolute error (MAE) for each existing model.

Model Name ^model number^	Mean (LCI, UCI)	Standard Error	MSE	MAE
De Aluja ^5^	−7 (−12.1, −1.9)	2.6	2168.5	2.2
Cherdchutham ^4^	30.1 (24.8, 35.3)	2.7	3148.0	2.7
Pearson ^2^	36.8 (32.4, 41.1)	2.2	2899.4	2.8
Horse weight tape	−31.6 (−40.9, −22.4)	4.7	7893.1	2.8
TDS Linear 2 ^10^	−28.4 (−34.4, −22.5)	3	3713.1	2.8
TDS Linear 1 ^9^	−28.7 (−34.7, −22.7)	3.1	3770.7	2.9
TDS Linear 3 ^11^	−33.8 (−39.8, −27.8)	3	4054.5	2.9
TDS Bayes ^12^	−34.3 (−40.3, −28.3)	3	4091.7	3
Kay ^8^	−43.5 (−49.7, −37.2)	3.2	5070.2	3.3
Eley ^1^	67 (62, 71.9)	2.5	6468.6	4.3
John (male) ^6^	−52.8 (−62.2, −43.3)	4.8	10,083.9	4.7
TDS GLM ^13^	104.1 (98.2, 110.1)	3	13,705.3	6.6
John (female) ^7^	−122.5 (−132.3, −112.6)	5	22,877.7	7.8
Donkey weight tape	−52.4 (−76.9, −27.9)	12.5	50,856.5	7.8
Noikhrua ^3^	−156.4 (−164.2, −148.7)	3.9	29,381.5	9.8

**Table 3:** Comparison of models predicting body weight (kg) recorded using a digital weighbridge. Model performance is evaluated using paired T-tests (T-value and *p*-value), Pearson correlation coefficients (r), and residual values (model weight subtracted from actual body weight). T-values indicate whether the model systematically overestimates (positive T-value) or underestimates (negative T-value) weight. The *p*-values assess statistical significance, with values < 0.001 indicating a significant difference from actual weights. The r values show the strength of the correlation between predicted and observed weights, and residual values indicate mean prediction errors, with positive values showing overestimation and negative values indicating underestimation.

Model Name ^model number^	T-Value	*p*-Value	r^2^	Residual
Cherdchutham ^4^	11.27	<0.001	0.89	30.08
TDS Bayes ^12^	−11.25	1	0.92	−34.28
TDS GLM ^13^	34.47	<0.001	0.88	104.12
TDS Linear 1 ^9^	−9.39	1	0.92	−28.73
TDS Linear 2 ^10^	−9.35	1	0.92	−28.44
TDS Linear 3 ^11^	−11.09	1	0.92	−33.79
De Aluja ^5^	−2.71	0.997	0.91	−7.04
Donkey weight tape	14.04	<0.001	0.81	39.93
Eley ^1^	26.69	<0.001	0.92	66.98
Horse weight tape	−10.28	1	0.92	−23.4
John (female) ^7^	−24.49	1	0.79	−122.47
John (male) ^6^	−10.96	1	0.79	−52.78
Kay ^8^	−13.69	1	0.91	−43.48
Noikhrua ^3^	−39.6	1	0.92	−156.42
Pearson ^2^	16.59	<0.001	0.92	36.77

**Table 4:** Hyperparameter tuning results of the XGBoost Model for Predicting weight of mules and hinnies. Metrics of the tuned XGBoost model, showing its predictive performance in estimating weight. The results include root mean squared error (RMSE), mean absolute error (MAE), R (coefficient of determination), and standard error, which collectively indicate the model’s accuracy and reliability.

Trees	Min *n*	Tree Depth	Learn Rate	Loss Reduction	Sample Size	Mean r^2^	*n*	Standard Error
1690	4	4	0.011	0	0.572	32.19	10	2.37
1445	19	3	0.005	0	0.746	32.22	10	2.32
134	6	9	0.034	0	0.418	32.46	10	2.15
1073	12	14	0.004	0	0.874	32.66	10	2.02
1567	3	8	0.008	0	0.934	32.67	10	2.42

## Data Availability

Data available from the corresponding author.

## Appendix A

**Table A1:** Summary statistics for various morphological parameters measured in mules and hinnies. For each parameter, the mean, 95% confidence interval (lower CI and upper CI), standard error, standard deviation, median, minimum, maximum, range, skewness, and kurtosis are provided. These statistics describe the central tendency, spread, and distribution shape of each measure, offering insight into the variability and normality of the dataset.

Parameter	Hybrid Type	*n*	Mean	Lower CI	Upper CI	S.E.	s.d.	Median	Variance	Min	Max	Range	Skew	Kurtosis
Back Length	Hinny	12	75.7	70.3	81.0	2.4	8.5	76.0	71.7	63.0	94.0	31.0	0.5	3.1
	Mule	304	85.0	83.9	86.1	0.6	9.7	84.0	93.2	51.0	109.0	58.0	−0.1	3.1
Body Length	Hinny	12	133.4	123.0	143.8	4.7	16.4	135.5	268.3	110.0	155.0	45.0	−0.1	1.5
	Mule	304	146.6	145.3	148.0	0.7	12.2	147.0	149.5	114.0	174.0	60.0	−0.2	2.6
Cannon Bone Girth	Hinny	12	17.1	15.8	18.4	0.6	2.1	17.8	4.4	14.0	20.0	6.0	−0.3	1.7
	Mule	304	18.7	18.5	19.0	0.1	2.2	19.0	4.9	13.5	33.0	19.5	1.8	12.6
Chest Width	Hinny	12	30.2	27.3	33.1	1.3	4.5	30.5	20.2	24.0	40.0	16.0	0.6	2.9
	Mule	304	36.0	35.5	36.6	0.3	4.7	36.0	21.9	21.0	50.0	29.0	0.0	3.2
Elbow Height	Hinny	12	73.2	62.4	84.0	4.9	17.0	74.8	288.8	28.0	90.0	62.0	−1.6	5.2
	Mule	304	85.2	84.4	86.0	0.4	7.1	86.0	50.4	44.0	99.0	55.0	−1.6	8.5
Forearm Girth	Hinny	12	30.6	27.7	33.5	1.3	4.6	29.8	20.8	25.5	43.0	17.5	1.7	5.7
	Mule	304	35.7	35.1	36.4	0.3	6.0	36.0	35.6	24.0	52.0	28.0	0.1	2.2
Head Length	Hinny	12	60.8	54.5	67.2	2.9	10.0	59.0	100.0	48.0	78.0	30.0	0.3	1.8
	Mule	304	65.8	65.0	66.6	0.4	6.9	66.0	47.8	45.0	85.0	40.0	−0.1	3.1
Head and Neck	Hinny	12	129.8	117.1	142.5	5.8	20.0	127.0	401.2	103.0	165.0	62.0	0.3	1.9
	Mule	304	135.4	133.8	137.0	0.8	14.1	134.5	200.2	96.0	176.0	80.0	0.3	2.9
Heart Girth	Hinny	12	154.9	145.8	164.0	4.1	14.4	161.0	206.3	125.0	174.0	49.0	−0.7	2.5
	Mule	304	167.8	166.1	169.4	0.9	14.8	167.0	220.4	128.0	220.0	92.0	0.4	3.6
Height	Hinny	12	129.1	119.5	138.8	4.4	15.2	131.8	231.6	105.0	151.0	46.0	−0.3	1.8
	Mule	304	144.0	142.9	145.1	0.6	10.0	144.5	100.0	110.0	166.0	56.0	−0.6	3.9
Hock Height	Hinny	12	49.8	45.6	53.9	1.9	6.5	49.5	41.8	42.0	59.0	17.0	0.1	1.6
	Mule	304	54.6	54.1	55.0	0.2	4.3	55.0	18.3	41.0	64.0	23.0	−0.5	3.1
Knee Girth	Hinny	12	27.0	25.0	28.9	0.9	3.0	27.5	9.3	22.0	31.0	9.0	−0.3	1.8
	Mule	304	29.4	29.1	29.8	0.2	2.8	29.5	8.0	22.0	38.5	16.5	0.3	3.5
Knee Height	Hinny	12	39.9	36.1	43.6	1.7	5.9	39.5	34.8	30.0	49.0	19.0	−0.1	2.0
	Mule	304	44.9	44.4	45.4	0.3	4.4	45.0	19.3	30.0	87.0	57.0	2.4	30.0
Neck Length	Hinny	12	69.0	61.0	77.0	3.6	12.6	67.0	158.7	55.0	96.0	41.0	0.7	2.6
	Mule	304	69.6	68.5	70.7	0.6	9.7	68.5	93.7	44.0	96.0	52.0	0.3	2.5
Rump Height	Hinny	12	130.1	120.0	140.2	4.6	15.9	130.5	252.3	106.0	154.0	48.0	−0.1	1.7
	Mule	304	145.5	144.4	146.6	0.6	10.0	147.0	100.6	111.0	169.0	58.0	−0.8	4.0
Rump Length	Hinny	12	43.1	36.7	49.5	2.9	10.1	41.0	102.3	31.0	60.0	29.0	0.5	1.9
	Mule	304	45.4	44.5	46.2	0.4	7.4	44.0	55.3	30.0	69.0	39.0	0.7	3.1
Shoulder Length	Hinny	12	47.5	43.0	52.1	2.1	7.2	47.5	51.7	34.0	61.0	27.0	0.0	2.7
	Mule	304	52.9	52.3	53.6	0.3	5.5	53.0	29.9	38.0	72.0	34.0	0.1	3.0
Donkey Weight Tape	Hinny	12	336.9	284.7	389.1	23.7	82.2	367.0	6751.7	180.0	457.0	277.0	−0.4	2.2
	Mule	304	331.8	313.5	350.1	9.3	160.9	380.0	25,885.0	0.0	540.0	540.0	−1.2	3.2
Horse Weight Tape	Hinny	12	298.9	250.1	347.8	22.2	76.9	318.0	5909.7	165.0	455.0	290.0	0.2	2.8
	Mule	304	355.7	345.6	365.7	5.1	89.0	356.0	7929.7	0.0	603.0	603.0	−0.4	5.5
Baody Weight	Hinny	12	305.5	254.0	356.9	23.4	81.0	328.5	6567.7	175.5	480.0	304.5	0.4	3.0
	Mule	304	387.8	376.2	399.3	5.9	102.6	376.0	10,524.8	179.0	739.0	560.0	0.5	3.0
